# Combined treatment of nerve growth factor and transcranical direct current stimulations to improve outcome in children with vegetative state after out-of-hospital cardiac arrest

**DOI:** 10.1186/s13062-023-00379-5

**Published:** 2023-05-10

**Authors:** Antonietta Curatola, Benedetta Graglia, Giuseppe Granata, Giorgio Conti, Lavinia Capossela, Luigi Manni, Serena Ferretti, Daniela Di Giuda, Domenico Marco Romeo, Maria Lucia Calcagni, Marzia Soligo, Enrico Castelli, Marco Piastra, Flavio Mantelli, Giacomo Della Marca, Susanna Staccioli, Tiziana Romeo, Marcello Pani, Fabrizio Cocciolillo, Aldo Mancino, Antonio Gatto, Antonio Chiaretti

**Affiliations:** 1 Dipartimento di Pediatria, Fondazione Policlinico Universitario “A. Gemelli” IRCCS, Rome, Italy; 2 Dipartimento di Pediatria, Università Cattolica del Sacro Cuore, Rome, Italy; 3 Istituto di Neurologia, Fondazione Policlinico Universitario “A. Gemelli” IRCCS, Rome, Italy; 4 Dipartimento di Scienze dell’Emergenza, Anestesiologiche e Rianimazione, Terapia Intensiva Pediatrica, Fondazione Policlinico Universitario “A. Gemelli” IRCCS, Rome, Italy; 5 Istituto di Farmacologia Traslazionale, Consiglio Nazionale delle Ricerche (CNR), Rome, Italy; 6 UOC di Medicina Nucleare, Fondazione Policlinico Universitario “A. Gemelli” IRCCS - Università Cattolica del Sacro Cuore, Rome, Italy; 7 Unità di Neurologia Pediatrica, Fondazione Policlinico Universitario “A. Gemelli” IRCCS, Rome, Italy; 8 Dipartimento di Neuroriabilitazione Intensiva, Ospedale Pediatrico “Bambino Gesù”, Rome, Italy; 9 Dompé Farmaceutici Spa, Via Campo di Pile, snc, L’Aquila, 67100 Italy; 10 Dipartimento di Scienze dell’Invecchiamento, Neurologiche, Ortopediche e della Testa-Collo, Fondazione Policlinico Universitario Agostino Gemelli, IRCCS, Rome, Italy; 11 Direttore Farmacia Policlinico Universitario Agostino Gemelli, IRCCS, Rome, Italy; 12 UOC di Medicina Nucleare, Fondazione Policlinico Universitario Agostino Gemelli IRCCS, Rome, Italy

**Keywords:** Human-recombinant nerve growth factor, Intranasal Administration, Neuroprotection, Transcranial Direct current stimulations, Out-Off Hospital Cardiac arrest

## Abstract

**Background:**

Out-of-hospital cardiac arrest (OHCA) is one of the most dramatic events in pediatric age and, despite advanced neurointensive care, the survival rate remains low. Currently, no effective treatments can restore neuronal loss or produce significant improvement in these patients. Nerve Growth Factor (NGF) is a neurotrophin potentially able to counteract many of the deleterious effects triggered by OHCA. Transcranial Direct Current Stimulation (tDCS) has been reported to be neuroprotective in many neurological diseases, such as motor deficit and cognitive impairment. Children with the diagnosis of chronic vegetative state after OHCA were enrolled. These patients underwent a combined treatment of intranasal administration of human recombinant NGF (hr-NGF), at a total dose of 50 gamma/kg, and tDCS, in which current intensity was increased from zero to 2 mA from the first 5 s of stimulation and maintained constant for 20 min. The treatment schedule was performed twice, at one month distance each. Neuroradiogical evaluation with Positron Emission Tomography scan (PET), Single Photon Emission Computed Tomography (SPECT), Electroencephalography (EEG) and Power Spectral Density of the brain (PSD) was determined before the treatment and one month after the end. Neurological assessment was deepened by using modified Ashworth Scale, Gross Motor Function Measure, and Disability Rating Scale.

**Results:**

Three children with a chronic vegetative state secondary to OHCA were treated. The combined treatment with hr-NGF and tDCS improved functional (PET and SPECT) and electrophysiological (EEG and PSD) assessment. Also clinical conditions improved, mainly for the reduction of spasticity and with the acquisition of voluntary finger movements, improved facial mimicry and reaction to painful stimuli. No side effects were reported.

**Conclusions:**

These promising preliminary results and the ease of administration of this treatment make it worthwhile to be investigated further, mainly in the early stages from OHCA and in patients with better baseline neurological conditions, in order to explore more thoroughly the benefits of this new approach on neuronal function recovery after OHCA.

**Supplementary Information:**

The online version contains supplementary material available at 10.1186/s13062-023-00379-5.

## Introduction

Out-of-hospital cardiac arrest (OHCA) is one of the most significant problems in industrialized countries [1–3]. The incidence of pediatric OHCA has been reported to be 8.0–10.6 per 100,000 children per year [4]. Although the survival rate of pediatric OHCA varies between age groups and the reasons for cardiac arrest, it remains very low (reported as 6.4 − 8%) [5, 6], depending on patient age, time of resuscitation and initial cardiac rhythm [4]. Children suffering from OHCA show long-term sequelae, more pronounced in behavioral, neurological and neuropsychological functions, that influence their quality of life [7] with a significantly negative impact in healthcare costs and emotional burden of family members [8]. The brain damage of OHCA-patients depends on the primary injury by the direct insult on neuronal, glial and vascular cells, and the secondary injury, which may include hypoxia, re-perfusion, edema, and diffuse ischemia [9] shaping the extent and severity of the cell impairment [10]. Secondary brain damage frequently causes the loss of cerebral neurons and can be associated with a progressive decrease in cognitive and motor functions. Targeting some of these secondary injury processes can play a pivotal role in the prevention or control of damage-induced cell death and brain tissue degeneration. Currently, no effective therapies can restore neuronal loss or produce significant improvement in these patients. Some advanced treatments, such as therapeutic hypothermia [11], neurotrophic factor administration and transcranial direct current stimulation (tDCS), have been proposed to improve the outcome of patients with brain injuries [12, 13], but the effects of such treatments has not been yet adequately established and evaluated. tDCS is a non-invasive cerebral stimulation method that acts by providing a direct current from a generator through electrodes placed on the scalp [12]. tDCS have been applied in adult patients with different neurologic impairment, such as deficit of memory, motor deficit to upper and lower extremity and difficulty in swallowing [13–17]. tDCS has also been tested as an option for a wide amount of different pediatric neurologic disorders, such as attention deficit and cognitive impairment [18–20]. Nerve Growth Factor (NGF) is the first discovered and most studied neurotrophin, with biological activities on central and peripheral nervous systems. The potential therapeutic applications of this neurotrophin have held for decades the promise to support neuronal growth, differentiation and survival of brain cells and neuronal sprouting [21, 22]. The ability of NGF to restore the function of injured neurons suggests that it may also counteract the deleterious neurologic damages triggered by OHCA.

NGF also regulates the glial response [23] both by arresting the cell cycle of astrocytes and therefore the process of reactive astrogliosis [24], and by stimulating the anti-inflammatory phenotype of microglia and blocking the release of pro-inflammatory cytokines [25]. NGF also stimulates angiogenesis and vasculogenesis [26], by inducing the production of vascular-endothelial growth factor (VEGF) [27–29], and supporting the proliferation and migration of endothelial cells [30]. Despite the promising potential of a NGF treatment, the poor permeability of the blood–brain barrier, when NGF is injected systemically [31], and the invasiveness of intraventricular administration, represented major obstacles to the development of NGF-based treatment to test in clinical trials targeting the central nervous system (CNS). Over the years, strategies for an effective NGF delivery to the brain have been investigated. Among the possible routes of administration of neurotrophins and other drugs targeting the CNS, intranasal delivery represents a non-invasive and easily accessible approach, which, however, is largely dependent on the specific physico-chemical and structural characteristics of each drug [32]. In preclinical studies, intranasal administration of NGF has shown its localization to reach the cerebral parenchyma, mainly by diffusing in the perivascular and perineural spaces of the olfactory and trigeminal nerves [33, 34]. In line with this, and suggesting an additional route, it has also been shown in rodents that ocular administration of NGF allows to reach basal ganglia and septum by retrograde transport through the optic nerves and optic pathways [35].

Given intranasally in rodents, NGF has been shown to reduce brain edema and improve motor and neurocognitive functions in rats with experimental induced brain hypoxia [36, 37]. Preclinical studies, performed by Dompé Farmaceutici Spa, confirmed that NGF delivered intranasally in mice and rats reaches the brain with preferential distribution to thalamus and hypothalamus (data not shown). Pre-clinical studies on the effect of intranasal NGF administration have been carried out in an open-head model of experimental traumatic brain injury (TBI) [36, 38, 39], in which NGF improved β-amyloid deposition and aquaporins-4-induced oedema and lowered tau phosphorylation in injured rat brain. Intranasal NGF treatment has only been previously attempted in two children with severe motor and cognitive impairment after TBI and meningitis, respectively, both showing an improvement in their clinical and neurological functions [40, 41]. According to experimental evidences and based on our previous experiences we report, for the first time ever, the effects of combined treatment of intranasal human-recombinant NGF (hr-NGF) and tDCS on brain functions of three children with chronic vegetative state [42] secondary to OHCA.

## Materials and methods

### Study design and eligibility

This is a prospective interventional study. Children affected by chronic vegetative state [42], secondary to OHCA, aged between 1 and 3 years were enrolled. We included only children whose deficits were stabilized, not responding to any treatment in place and have a clinical, neuroradiological and instrumental documentation that supported the diagnosis of a vegetative state. The written informed consent signed by parents was required for enrollment of the patients. The study was conducted in accordance with Good Clinical Practice, the Declaration of Helsinki and our local hospital regulations.

### Study procedures

Children underwent intranasal administration of hr-NGF (Oxervate by Dompè Farmaceutici, Milan, Italy) associated with tDCS in two cycles of ten days each. Neurological evaluation with instrumental examinations such as Magnetic Resonance Imaging (MRI), Positron Emission Tomography scan (PET), Single Photon Emission Computed Tomography (SPECT), Electroencephalography (EEG) and Power Spectral Density (PSD) was determined before the first cycle of treatment and one month after the end of the second one. The primary outcome was to identify any evidence of changes in clinical and neurological conditions and, also, modifications in neuroradiological findings and electroencephalographic patterns after the treatment. The secondary outcome was to study the safety and the tolerability of the treatment.

#### Intranasal hr-NGF administration

Each patient received intranasal clinical grade hr-NGF aqueous solution at a dose of 50 gamma/kg. For treatment by intranasal administration, the commercial product OXERVATE, consisting of cenegermin-bkbj (rhNGF) ophthalmic solution 0.002% (20 mcg/mL) was used (Dompè Farmaceutici SpA, Milano, Italy).

This total amount was administered infusing a defined amount of the product for each nostril three times daily over 10 days, according to the total amount required (defined by the weight of each patient). This treatment schedule will be performed twice, at one month distance each. The hr-NGF was administered directly into the nostril by Mucosal Atomization Device (MAD), used to deliver medication via a fine spray (30 µicron) to facilitate the absorption of the drug.

This amount is considered sufficient to reach and stimulate NGF receptors, mainly Tyrosine receptor kinase A (TrkA), in most cerebral cholinergic and serotoninergic areas of the brain, as previously reported in literature [43, 44]. Before NGF administration nostrils were washed with 1 mL of saline solution, successively aspirated, to avoid any interference with the drug absorption.

#### Transcranial direct current stimulation

Each patient underwent to anodal tDCS of left dorsolateral prefrontal cortex (DLPF). tDCS was performed with a constant current electrical stimulator (NeuroConn, DC Stimulator MC), by using saline-soaked surface sponge electrodes (5 × 7 cm). The anode (stimulating electrode) was positioned over the left DLPF cortex (F3, according to the 10–20 international system for EEG placement) while the cathode (i.e., reference electrode), was placed over the contralateral shoulder.

During tDCS, the current intensity was increased from zero to 2 mA from the first 5 s of stimulation and maintained constant for 20 min. At the end of stimulation the current was ramped down from 2 mA to 0 in 5 s before stopping the stimulation. Impedances were kept always under 10 kΩ. The same protocol was applied each day, for 10 consecutive days.

#### Neurological assessment

The neurological assessment was deepened by using modified Ashworth Scale and the Gross Motor Function Measure (GMFM) **(eFigures 1 and 2)**, performed before the first cycle and after the second one. The modified Ashworth Scale is the most commonly accepted clinical tool used to measure the increase of muscle tone and to determine the efficacy of treatments in patients with spasticity [45, 46].

The GMFM is an observational clinical tool designed to quantitatively evaluate changes in gross motor function in children with cerebral palsy [47]. The GMFM shows sufficient validity, responsiveness, and reliability for assessment of motor skills of children with cerebral palsy undergoing rehabilitation. Infants were also classified according to the Gross Motor Function Classification System (GMFCS) to classify each child’s level of gross motor function with skill levels from I to V, assessing the children’s gross motor function by observation [48] (eFigure 3). In addition, the study aims to detect the presence of changes in their quality of life measured by the Disability Rating Scale **(DRS, eFigure 4)** to assess the clinical conditions before the first and after the second cycle of therapy, with the collaboration of the parents/caregivers. The DRS was developed as a method of rating the effects of traumatic brain injury to determine how long recovery might take [49].

### Safety assessment

Patients were assessed by medical history and physical examination at baseline, at the beginning of the second cycle of therapy and one month after the end of the treatment. Safety was evaluated by the analysis of adverse events and symptoms connected to study medication. Every clinical change during the administration of hr-NGF and tDCS was recorded. The investigator and the parents of the child evaluated treatment tolerability.

#### Neuroradiological assessment

The neurological assessment in all treated children was highlighted by using PET, SPECT, EEG, and PSD. For all technical details of these procedures see Supplementary Materials.

## Results

**Fig. 1:**
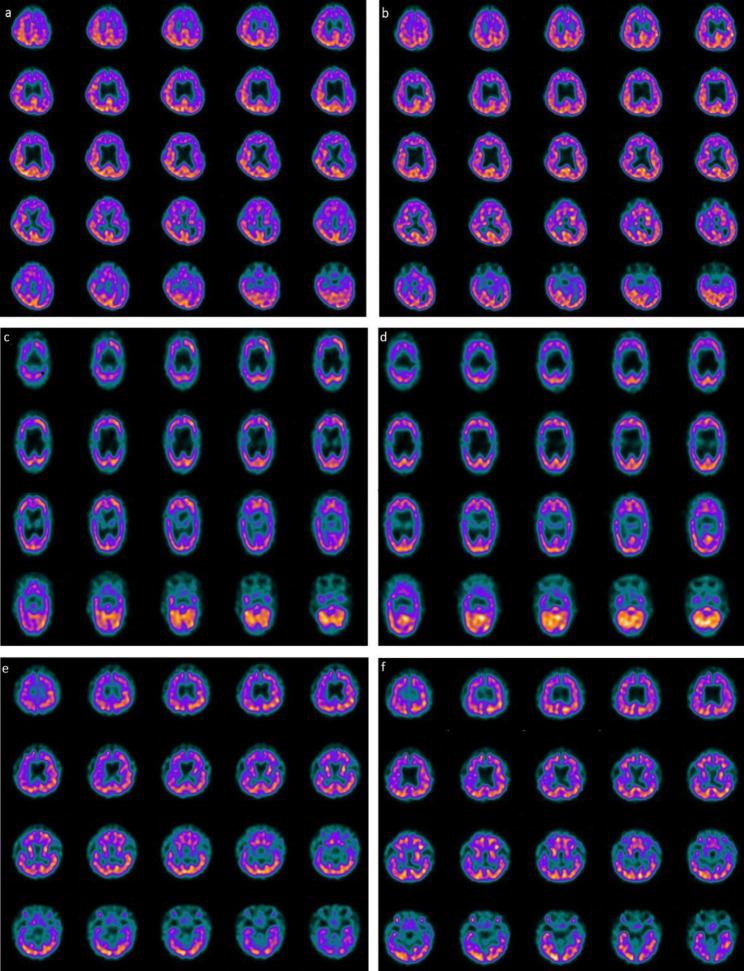
Perfusion SPECT images before and after combined treatment with hr-NGF and tDCS. **(a)**^99m^Tc-HMPAO SPECT images (transaxial slices) of the first patient, before the combined treatment, showed a moderate reduction in radiotracer uptake (hypoperfusion) in the left frontal and temporal cortices, as well as in the left caudate nucleus, left putamen and right thalamus. **(b)** After the combined treatment, a mild increase in ^99m^Tc-HMPAO uptake was detected in the left frontal cortex (+ 10%), left temporal cortex (+ 9%), left caudate nucleus (+ 17%), left putamen (+ 13%) and right thalamus (+ 12%). **(c)** ^*99m*^Tc-HMPAO SPECT images (transaxial slices) of the second patient, before the combined treatment with hr-NGF and tDCS, showed a moderate-to-severe reduction in radiotracer uptake (hypoperfusion) in the right and left frontal cortices, right and left parietal cortices, left occipital cortex, caudate nucleus, putamen and thalamus, bilaterally. **(d)** After the combined treatment, a slight increase in radiotracer uptake was detected in the right frontal cortex (+ 6%), left parieto-occipital cortex (+ 8%) and cerebellum (+ 11%). **(e)** In the third patient, ^99m^Tc-HMPAO SPECT images (transaxial slices), before the combined treatment with hr-NGF and tDCS, showed a mild reduction in radiotracer uptake (hypoperfusion) in the right and left frontal cortices, right parietal cortex, right and left temporal poles, in the thalami and cerebellar hemispheres. **(f)** After the combined treatment, a slight increase in radiotracer uptake was detected in the right and left frontal cortices (7+% and + 8%, respectively), right parietal cortex (+ 7%), right temporal pole (+ 11%) and cerebellum (+ 7%) (b)

**Fig. 2:**
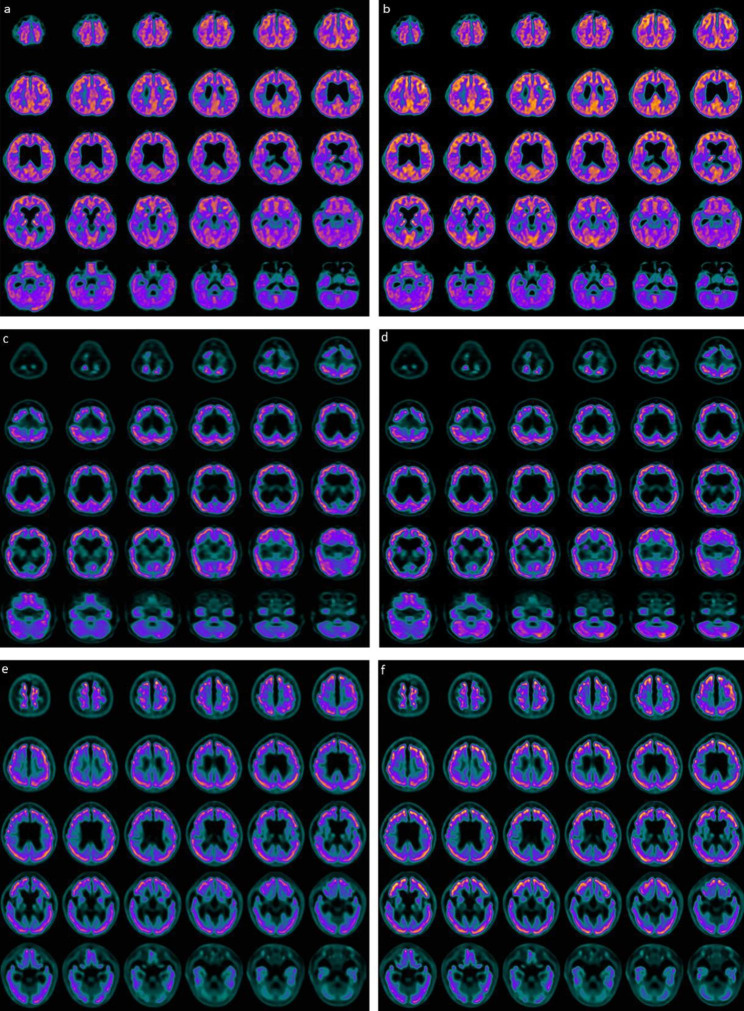
Brain 18 F-FDG PET axial slices before and after combined treatment with hr-NGF and tDCS. **(a)** In the first patient, before the combined treatment, a moderate global reduction in 18 F-FDG uptake was observed in all cortical regions, while radiotracer uptake was slightly decreased in all subcortical areas. **(b)** After the combined treatment, an increase in radiotracer uptake was detected in the right and left frontal cortices (right: +11%; left: +12%), right and left parietal cortices (right: +26%; left: +25%), right and left occipital cortices (right: +26%; left: +27%). **(c)** Brain 18 F-FDG PET axial slices of the second patient, before the combined treatment with hr-NGF and tDCS, showed a severe global reduction in 18 F-FDG uptake in all cortical and subcortical regions, more pronounced in the latter. **(d)** After the combined treatment, an increase in radiotracer uptake was detected in the caudate nucleus, bilaterally (right: +51%; left: +51%) and cerebellum (+ 20%). **(e)** In the third patient, brain 18 F-FDG PET axial slices before the combined treatment showed a mild global reduction in 18 F-FDG uptake in all cortical regions and a more severe reduction in all subcortical regions. **(f)** After the combined treatment, an increase in radiotracer uptake was detected in the bilateral frontal cortex (right: +29%; left: +34%), bilateral parietal cortex (right: +15%; left: +19%), right and left occipital cortices (right: + 20%; left: +19%) and cerebellum (+ 14%)

In this study we included three children suffering from a chronic vegetative state secondary to OHCA. Two of them (66.7%) were male; their mean age at the time of enrollment was 36 months. The average time between the brain damage suffered and the start of combined treatment with hr-NGF and tDCS was 32 months (range 26–36). All children enrolled were hospitalized in the first two months of life in pediatric intensive care unit (PICU) after a cardiac arrest, following milk inhalation in two cases and of unknown etiology in another. Their Glasgow Coma Scale (GCS) at the admission in PICU was 4. All children after OHCA presented severe neurological outcomes with dysphagia, inability to speech, reflex movements without response to command, and minimally conscious state in the presence of a vegetative state. All underwent tracheostomy for continuous mechanical ventilation and gastrostomy for enteral nutrition. In all cases, cerebral MRI scan showed severe damages to the grey matter, especially involving the basal ganglia, thalamus and brain stem, secondary to acute and severe hypoxia in children. This diffuse loss of brain tissue was strongly associated to a compensatory enlargement of the cerebrospinal fluid (CSF) spaces resulting in hydrocephalus ‘ex vacuo’ (data not shown). At the time of enrolment all children presented a mean Glasgow Outcome Scale (GOS) of 2. Neurological evaluation showed a vegetative state with psychomotor delay, areflexia, dysphagia, diffuse and severe limb hypertone and tendency to hypothermia and bradicardia. All patients were subjected to PET, SPECT, EEG, PSD and clinical neurological examination before and after the combined treatment, according to the same schedule. In all three patients, first SPECT and PET pointed out a marked and global reduction in tracer uptake at the cortical, subcortical and cerebellar levels **(**Figs. 1 and 2). After the two cycles of treatment, in all cases SPECT and PET showed a marked increase in tracer uptake in specific brain areas. All detailed descriptions of neuroimaging are reported in Figs. 1 and 2.

**Fig. 3:**
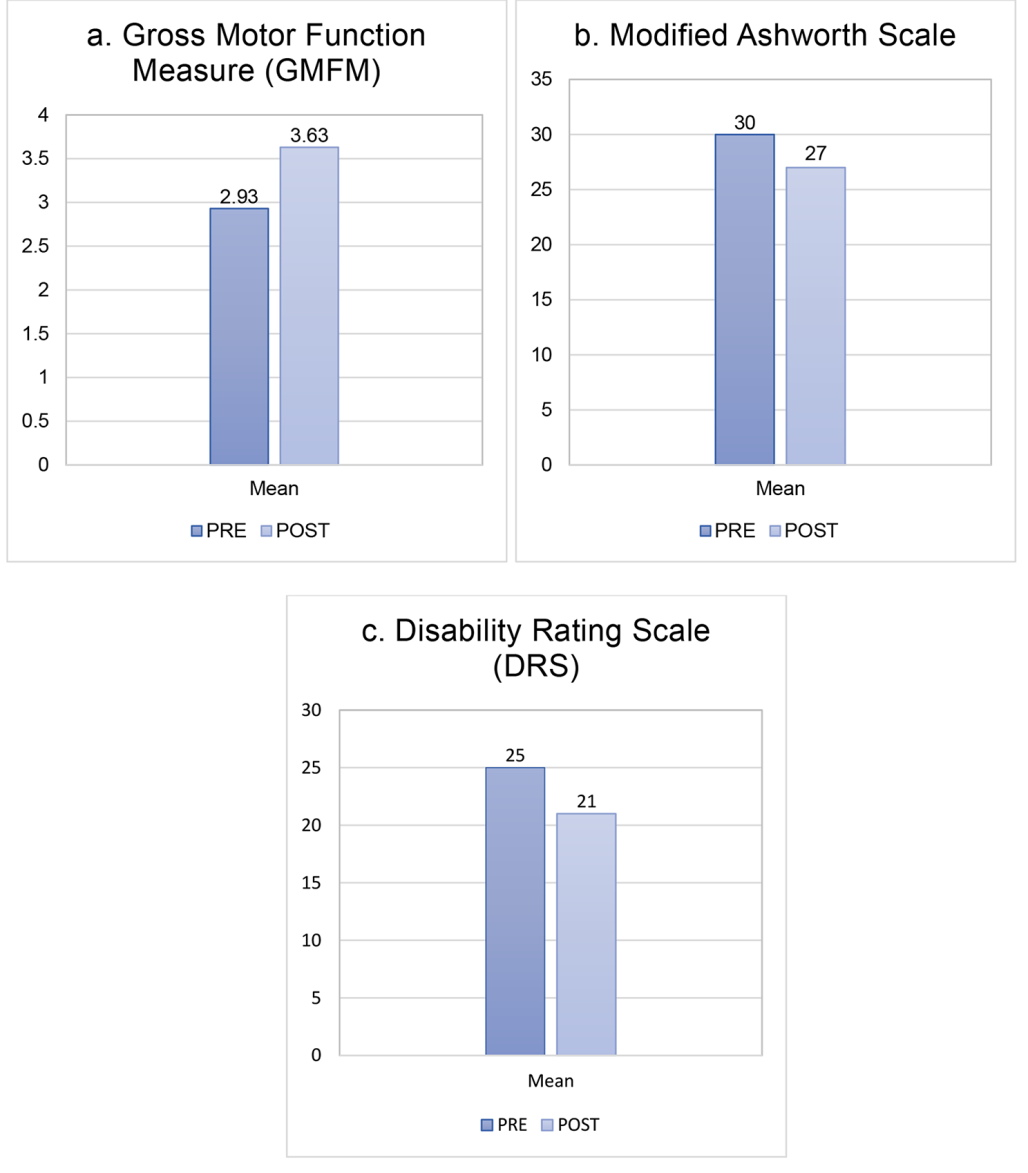
Changes of the clinical scales used to detect the improvement of children after the treatment with hr-NGF and tDCS. **(a)** A mean improvement of 23% of GMFM (from 2.93–3.63%) was recorded after the treatment. **(b)** A mean improvement of 3 points on modified Ashworth Scale showed a post treatment reduction of hypertonus. **(c)** An improvement of 4 points in DRS was observed in all treated patients

In addition, the clinical neurological assessment showed a reduction in involuntary movements on mobilization and a slight increase in polydistrict joint excursion. Some passive facial brushing movements and semi-sitting posture for prolonged periods were possible. In addition, all children showed the acquisition of voluntary movements of their fingers, improved facial mimicry with opening of the eyes, small grimaces, little smiles, emission of sounds, and reaction to painful stimuli. The mean GMFM pre-treatment was 2.93% (V level for GMCS for infant cerebral palsy). An improvement of GMFM in a range from 18 to 28% was evidenced after the treatment **(**Fig. 3a**).** An average improvement in spasticity (assessed by modified Ashworth Scale) of 3 points was reported, with progressive improvement of muscle hypertonus **(**Fig. 3b**).** For all patients, an improvement in muscle spasticity, facial expressiveness and responsiveness was also documented by caregivers. In fact, according to the DRS, all three patients collected an initial score suggestive of an extreme vegetative state (25–29 points). At the end of the treatment, there was a mean improvement of 4 points in their DRS, with a consequent change of category from Extreme Vegetative State to Severe Disability (17–21 points) **(**Fig. 3c**).**

**Fig. 4:**
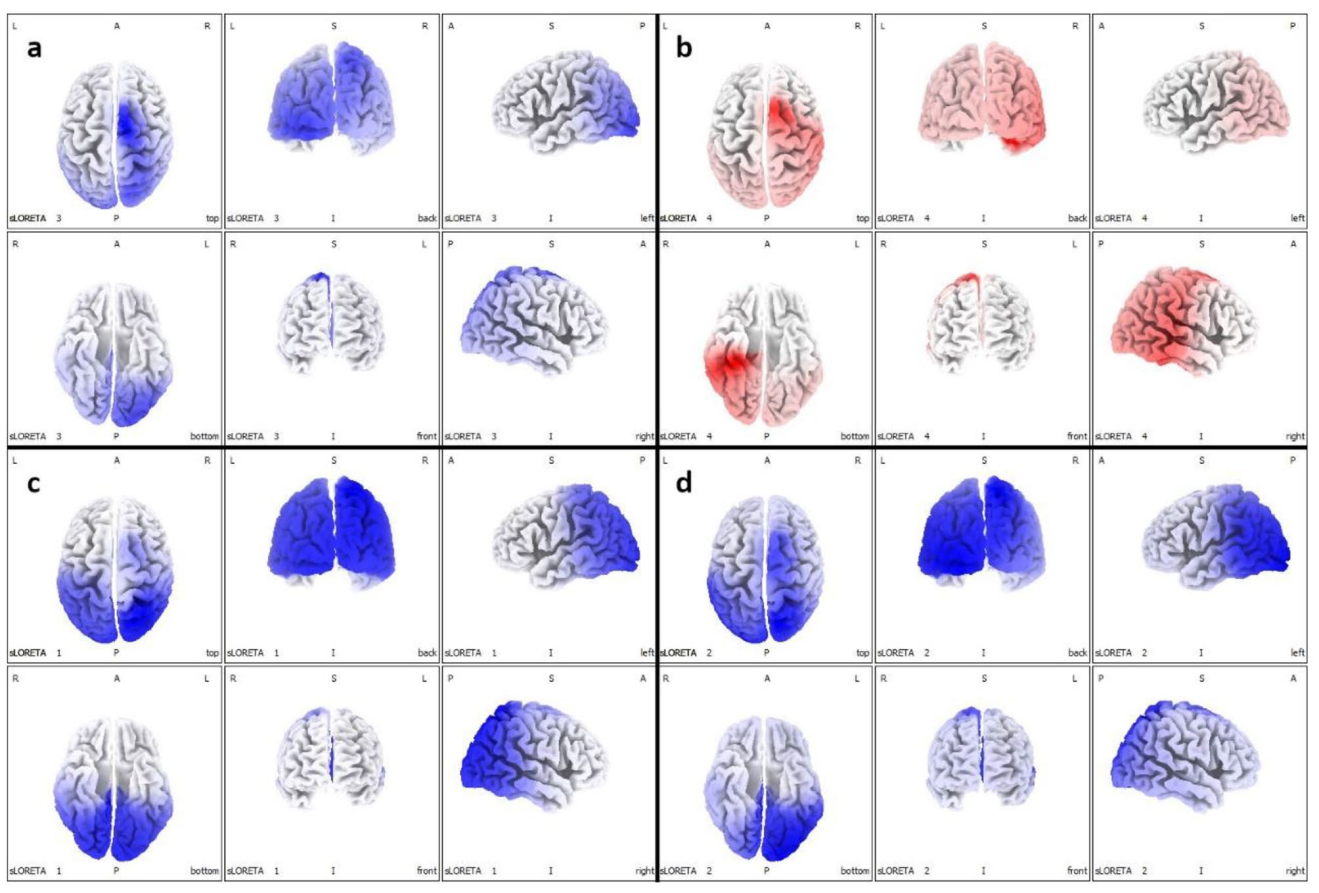
Topographical representation of the Power Spectral Density distribution of EEGs signals. The modifications of PSD show a mild reduction of slow frequency alpha band in medial frontal gyrus (Brodmann area 6) and cingulate gyrus (Brodmann area 24), bilaterally **(a)**. Considerable reduction of slow frequency bands delta in superior parietal lobule and precuneus (Brodmann area 7) bilaterally **(c)**, and theta in superior parietal lobule (Brodmann area 7), fusiform and lingual gyrus (Brodmann area 18), bilaterally **(d)**. Increase signals in the fast band activities beta especially in limbic lobe and hippocampal gyrus (Brodmann areas 20, 35 and 36) in right hemisphere **(b)**

Topographical analysis of the PSD distribution of the EEG signal documented a reduction in the PSD of the slow frequency bands (delta and theta) in post treatment records, a more modest reduction in the PSD of the alpha band, and an increase in the fast band activities (beta). These changes had a different distribution, as highlighted in Fig. 4. No adverse effects were reported during the study period.

## Discussion

Our study reports, for the first time to our knowledge, the effects of combined treatment with intranasal hr-NGF and tDCS in three children affected by chronic vegetative state after prolonged OHCA. The devastating neurologic sequelae caused by cardiac arrest and associated cerebral hypoperfusion have been recognized since the early development of modern resuscitation techniques [50]. The persistence of unfavorable outcome after years from the event, despite advances in cardio-pulmonary resuscitation, led the American Heart Association to recognize brain injury after cardiac arrest as an important area for both clinical and experimental researches [51]. Global cerebral hypoxia and ischemia after OHCA results in heterogeneous injury to the brain. Large projection neurons of the cerebral cortex, cerebellar Purkinje cells, and the CA-1 area of the hippocampus are the most vulnerable sites [52], while the subcortical areas, such as the brain stem, thalamus, and hypothalamus, are more resistant to injury [53]. To date, there are no therapeutic or pharmacologic approaches to improve neurologic outcome in this kind of patients. Despite advanced and up-to-date critical, neuro-intensive and rehabilitative care, our patients showed no neurological improvement about 30 months after OHCA, so we decided to start an experimental combined treatment with intranasal hr-NGF administration and tDCS. This new therapeutic approach was followed by a relevant improvement of functional (PET/CT and SPECT/CT) and electrophysiological (EEG and PSD) results, with a concomitant amelioration of the patients’ clinical and neurological conditions. In fact, after this combined treatment, all children showed the acquisition of voluntary movements of their fingers, improved facial mimicry with facial brushing movements, small grimaces and little smiles, semi-sitting posture for prolonged time, with consequent better interaction with their parents and caregivers, as testified by the changes in GMFM and DRS. Moreover, the most important clinical improvement to highlight in all patients is the significant reduction of their muscle spasticity, as reported by the modifications of Ashworth Scale. Following the treatment with hr-NGF and tDCS a marked amelioration in the muscle hypertone was evidenced, with a progressive reduction of their spasticity. This result correlates well with the increased uptake in glucose metabolism and vascularization in the cerebellum and thalamus bilaterally and with the improvement of brain perfusion in the same areas. Both these changes have been documented by PET and SPECT investigations at the level of the two aforementioned structures and by the improvement in brain electric activity documented via PSD at the level of Brodmann’s areas, which is in communication with the thalamus. To this regard, it is important to mention that muscle tone is closely related to these two structures: on one hand the cerebellum which actively participate in the regulation of posture and muscle tone and, on the other hand, the thalamus which is in communication with the premotor and supplementary motor cortex (Brodmann area 6), with an established connection to both voluntary and non-voluntary movements. The extensive role of neuroprotective effects of NGF, in both central and peripheral nervous systems, has already been widely reported, mainly as part of neuronal damage caused by OHCA [54, 55]. Delivering neurotrophic factors into the brain has classically been a significant challenge owing to the presence of the blood–brain and blood–cerebrospinal fluid barriers, limiting the drug penetration into the CNS [36, 56]. Accumulated experiences have pointed to the existence of a direct pathway from the nose to the brain that can facilitate the release of drugs into the CNS. Although the exact mechanisms are not completely understood, growing evidences suggest that these pathways involve olfactory nerve terminations, trigeminal nerve fibers, vascular and lymphatic pathways, that allow NGF to be actively transported into the whole cerebral parenchyma [34, 57, 58]. In experimental animal models, intranasal NGF administration showed different actions after severe OHCA, such as attenuating amyloid β42 deposits and brain edema, inhibiting the transcription and expression of pro-inflammatory cytokines, minimizing mitochondria-mediated apoptosis and, finally, reducing the elevation of interleukin 1β and other pro-inflammatory cytokines [38, 39]. Intranasal NGF, delivered to rat brain parenchyma, reaches the olfactory bulb, cortex, cerebellum, brain stem, hippocampus, amygdala and striatal level in significantly high concentrations [59]. At the striatal level, one of the possible targets of NGF could be cholinergic interneurons expressing TrkA receptor in detectable amounts, both during development and in adulthood [60, 61]. Alterations of brain cholinergic neurons have been reported as a consequence of brain trauma and ischemia [62–64], and striatal cholinergic dysfunction is associated with various forms of dystonia and spasticity [65], which are a common long-term neurological sequelae in patients suffering from chronic vegetative state after OHCA [66]. It can therefore be hypothesized that NGF improves motor behaviour in these patients by acting directly on NGF-responsive striatal neuronal populations or indirectly by regulating the striatal microenvironment, improving OHCA-induced astrogliosis, and limiting the increase in tissue GFAP content [24]. tDCS has been reported to be neuroprotective in many experimental neurological diseases, by reducing caspase-3 positive neurons after ischemia/reperfusion injury and by alleviating cerebral inflammation and neuronal apoptosis. Recently, it has also been reported that Brain-Derived Neurotrophic Factor (BDNF) signaling pathway in the cerebral penumbra area is upregulated, suggesting that BDNF/TrkB signals and their downstream PI3K/Akt signaling pathway play a pivotal role in electrical stimulation-related neuroprotection [67]. Applying anodal tDCS, over the rodent hippocampus/prefrontal cortex, enhanced BDNF expression in the stimulated brain regions, so improving cognitive function in rat brain [46]. tDCS influences neuronal plasticity and cognitive functions not only involving thalamus-cortical structures, by the restoring synapses plasticity through the repolarization of the NMDA receptors [12]. They also up-regulating endogenous neurotrophic factors biosynthesis, further contributing to the improvement of upper and lower extremity spasticity, as showed in our patients and as previously reported in the literature [68–70]. These intriguing results obtained with this combined therapy open a new potential rescue treatment in children suffering from severe neurological impairment after OHCA. Neuroradiological, electrophysiological, and clinical changes observed in these children have been so remarkable and occurred in a relatively short time (about two months after the end of therapy) that is conceivable they are related to the neuroprotective effects of the combined treatment, rather than to a “spontaneous recovery” occurred after such a long time from the initial hypoxic-ischemic event.

In conclusion, our results are considered promising and will hopefully pave the way to further clinical research to evaluate the potential effectiveness of intranasal hr-NGF administration and tDCS for improving clinical outcome in children with severe neurological impairment after OHCA. The current results are, in fact, preliminary and will be supportive to the initiation of a full clinical development program in a well-controlled setting. Additional evidence may also come to support our current results, by a parallel ongoing open label clinical studies in children with severe traumatic brain injury, as well as from the formal toxicological and pharmacodynamic studies performed for regulatory purposes by Dompé Farmaceutici Spa.

The ease of administration of our combined treatment makes it certainly worthwhile to be investigated further, mainly in the early stages from OHCA and in patients with better baseline neurological conditions, in order to explore more thoroughly the benefits of this new therapeutic approach on cerebral function recovery in children with vegetative state after OHCA.

## Limitations of the study

Because of the low sample size our data may not be generalizable to all children with chronic vegetative state after OHCA so, to minimize the selection bias of patients, clear guidelines for the assessment and treatment of this kind of patients must be used, considering that is very difficult to know the actual duration of OHCA and the time elapsed from the start of cardiopulmonary resuscitation, with obvious repercussions on the outcome of these children. Moreover, due to the heterogeneity of stimulation protocols and lack of wider randomized clinical trials, more data on the efficacy and safety of tDCS are required to assess the impact of this technique on neurocognitive development and motor function recovery in children. Furthermore, understanding the right dose of NGF to utilize and also the administration schedule represents, even today, an important challenge to be faced. The difficulties in treating this kind of patients and the limitations of the techniques used to determine the efficacy of this combined treatment may limit the development of this type of study, but defining the relationships between hr-NGF administration and tDCS in the injured brain could allow the individualization of new strategies for the treatment of patients suffering from vegetative state after OHCA.

## Electronic supplementary material

Below is the link to the electronic supplementary material.

Supplementary Material 1

Supplementary Material 2

Supplementary Material 3

Supplementary Material 4

Supplementary Material 5

Supplementary Material 6

## Data Availability

N/A.

## Abbreviations

CNS: Central nervous system
CSF: Cerebrospinal fluid
DRS: Disability Rating Scale
DLPF: Dorsolateral prefrontal cortex
EEG: Electroencephalography
GCS: Glasgow Coma Scale
GOS: Glasgow Outcome Scale
GMFCS: Gross Motor Function Classification System
GMFM: Gross Motor Function Measure
hr-NGF: Human-recombinant NGF
MRI: Magnetic Resonance Imaging
MAD: Mucosal Atomization Device
NGF: Nerve Growth Factor
OHCA: Out-of-hospital cardiac arrest
PICU: Pediatric Intensive Care Unit
PET: Positron Emission Tomography scan
PSD: Power Spectral Density
SPECT: Single Photon Emission Computed Tomography
tDCS: Transcranial direct current stimulation
TBI: Traumatic brain injury
TrkA: Tyrosine receptor kinase A
VEGF: Vascular-endothelial growth factor
