# Supplementary material for: Combined treatment of nerve growth factor and transcranical direct current stimulations to improve outcome in children with vegetative state after out-of-hospital cardiac arrest
Source: Biol Direct. 2023 May 10;18:24. doi: 10.1186/s13062-023-00379-5 (PMC10170696; doi:10.1186/s13062-023-00379-5)
Supplement: Supplementary file 1 — Supplementary Material 1 [file 13062_2023_379_MOESM1_ESM.docx]

**SUPPLEMENTARY MATERIALS**

**^18^F-FDG PET-CT imaging**

Brain PET-CT scans before and after hr-NGF treatment were performed according to the following protocol: intravenous injection of 3 MBq/Kg of ^18^F-FDG in fasting state (6 hours), in normoglycemic conditions (<160 mg/dl) and in resting state in a quiet environment, with eyes closed. Forty minutes post-injection, each child was placed in the supine position in the scanner (Biograph mCT, Siemens Healthineers, Chicago, Illinois), using a head-holder to maintain the head in the same position. After a CT scout to define the brain axial imaging range, a CT scan (tube current 75 mA, voltage 120 kV, pitch 0.938, rotation time 0.5 sec) was carried out, followed by a 3D PET acquisition (lasting 20 minutes). CT was employed for attenuation correction of PET emission data. Matched CT and PET images were reconstructed using iterative methods; time-of-flight, attenuation-corrected PET images were visually analyzed in transverse, sagittal and coronal planes. Subsequently, a semi-quantitative analysis of PET data was performed using Statistical Parametric Mapping (SPM8, Wellcome Department of Imaging Neuroscience, Institute of Neurology, London, UK), through Volumes of Interests (VOIs) selected from a digital atlas and placed over the grey matter of both hemispheres and cerebellum. Using the MarsBaR toolbox, VOI mean activity concentration was extracted and expressed as a percentage of the global mean activity. For each child, the percentage differences in the mean activity concentration of selected VOIs between the two examinations were calculated. Detailed information about the PET/CT examination (including the increase in radiation exposure due to the CT scan) was given to the parents of each child, and they provided their written informed consent according to our Ethics Committee.

**^99m^Tc-HMPAO SPECT/CT imaging**

Brain Single Photon Emission Computed Tomography (SPECT) before and after hr-NGF treatment was performed using ^99m^Tc-hexamethyl-propylene-amine oxime (HMPAO) as the perfusion tracer. Sixty minutes after the intravenous administration of 12 MBq/Kg of ^99m^Tc-HMPAO (GE Healthcare, United Kingdom), each child underwent brain imaging, using a SPECT/CT hybrid system (Symbia Intevo 2, Siemens, Germany) equipped with high-resolution, low-energy parallel-hole collimators. The following acquisition parameters were employed: radius of rotation ≤ 15 cm, 120 projections over 360°, 128 x 128 matrix, zoom factor 1.23, 40 seconds per view. Data reconstruction was carried out using an iterative technique (OSEM Flash 3D™: 12 iterations with 12 subsets and a Gaussian smoothing filter, FWHM: 6 mm). A low-dose computed tomography (CT) image for attenuation correction was acquired, using a 2-detector row CT on the SPECT/CT scanner with a dose modulation technique (Siemens CARE Dose™). Attenuation-corrected SPECT images were visually analyzed in transaxial, sagittal and coronal planes. Afterwards, a semi-quantitative analysis of SPECT data was performed using Statistical Parametric Mapping (SPM8, Wellcome Department of Imaging Neuroscience, Institute of Neurology, London, UK), through Volumes of Interests (VOIs) selected from a digital atlas and placed over the grey matter of both hemispheres and cerebellum. Using the MarSbaR toolbox, VOI mean activity concentration was extracted and expressed as a percentage of the global mean activity. For each child, the percentage differences in the mean activity concentration of selected VOIs between the two examinations were calculated. Detailed information about the SPECT/CT examination (including the increase in radiation exposure due to the CT scan) was given to the parents of each child, and they provided their written informed consent according to our Ethics Committee.

**EEG:**

Continuous EEG recordings were performed during wake and during sleep. Periods of wake were detected visually, on the basis of the EEG trace and the simultaneous video recording. Epochs of EEG slowing, consistent with drowsiness, were identified visually and thus excluded from analysis. EEG was recorded by means of a Micromed System Plus digital EEGgraph (Micromed^©^ S.p.A., Mogliano Veneto, TV, Italy). EEG montage included 8 standard scalp leads positioned according to the 10 - 20 system (recording sites: Fp1, Fp2, T3, C3, C4, T4, O1, O2). The reference electrodes were placed on the linked mastoids. Impedances were kept below 5KΩ before starting the recording and checked again at the end.

In particular, impedances of the mastoids reference electrodes were checked to be identical. This electrode montage is considered as an adequate EEG spatial sampling for the estimation of cortical sources of eyes-closed resting state EEG rhythms with the eLORETA software, since these rhythms are widely represented across all human cerebral cortex in contrast to the circumscribed functional topography of event-related EEG changes (especially at high frequencies) in response to specific sensory or motor events. Therefore, eyes-closed resting state EEG rhythms can be properly sampled with a relatively low amount of electrodes, as opposed to the higher spatial sampling required to take into account the detailed functional topography of event-related EEG activity. This relatively low-spatial sampling of EEG rhythms is in line with the fact that LORETA solutions are intrinsically maximally smoothed at source space, due to its regularization procedure. Sampling frequency was 256 Hz; analogic to digital conversion was made at 16 bit; pre-amplifiers amplitude range was ±3200 µV and low-frequency pre-filters were set at 0.15 Hz. The following bandpass filters were used: HFF = 0.2 Hz; LFF = 128 Hz. The line noise (in Italy: 50Hz) was removed by using a 50Hz notch filter. In the off-line analysis, rejection of artifacts (eye movements, blinks, muscular activations, respiratory or movement artifacts) was performed visually on the raw EEG trace, by posing a marker at the onset of the artifact signal and a further marker at the end of the artifact. Successively, the artifact segment (that is, the EEG signal interval included between the two markers) was deleted, and this cancellation involved all the EEG traces acquired within that interval. In this way, all the EEG intervals characterized by the presence of artifacts were excluded from the analysis. After artifact rejection, the remaining EEG intervals were exported into American Standard Code for Information Interchange (ASCII) files, and imported into the eLORETA software. We analyzed segments of EEG recorded in wake and during NREM and REM. The average length of each EEG segment analyzed were the following: wake=371±23 sec.; NREM=418±12 sec.; REM=379±34 sec. Detailed method of PSG recording have been described previously. All EEG analysis were performed by means of the eLORETA software.

### eLORETA

For the estimation of cortical sources of EEG rhythms was used LORETA software as provided at [http://www.unizh.ch/keyinst/NewLORETA/LORETA01.htm](http://www.unizh.ch/keyinst/NewLORETA/LORETA01.htm%20). LORETA belongs to a family of linear inverse solution procedures modeling 3D distributions of [EEG](javascript:void(0);) sources. eLORETA computes 3D linear solutions for the EEG inverse problem within a three-shell spherical head model including scalp, skull, and brain compartments. LORETA solutions consist of voxel current density values able to predict [EEG](javascript:void(0);) power spectral density (PSD) at scalp electrodes. eLORETA is a reference-free method of EEG analysis, which allows to obtain the same source distribution for EEG data independently from the reference electrode. Estimated cortical sources of scalp [EEG](javascript:void(0);) voltages are expected to reflect the synchronous synaptic neural currents of the [pyramidal](javascript:void(0);) cortical neurons, which are associated to local field potentials. LORETA computes 3D linear solutions (LORETA solutions) for the EEG inverse problem within a three-shell spherical head model including scalp, skull, and brain compartments. The brain compartment is restricted to the cortical gray matter/hippocampus of a head model co-registered to the Talairach probability brain atlas and digitized at the Brain Imaging Center of the Montreal Neurological Institute. eLORETA images represent the standardized electrical activity at each of 6239 cortical voxels (spatial resolution 5 mm) in Montreal Neurological Institute space. LORETA computes relative currents for z, x, and y components of any dipole.

### Frequency analysis

EEG frequency analysis was performed by means of Fast Fourier Transform algorithm, with a 2 seconds interval on the EEG signal, in all scalp locations. The following frequency bands were considered: delta (0.5–4 Hz); theta (4.5–7.5 Hz); alpha (8–13.5 Hz); beta (14-30 Hz). For frequency analysis, monopolar EEG traces (each electrode referred to joint mastoids) were used. Topographic sources of EEG activities were determined using the eLORETA software. The eLORETA software computes the current distribution throughout the brain volume. In order to find a solution for the 3-dimensional distribution of the EEG signal, the eLORETA method assumes that neighbouring neurons are simultaneously and synchronously activated. This assumption rests on evidence from single cell recordings in the brain that shows strong synchronization of adjacent neurons. The computational task is to select the smoothest of all possible 3-dimensional current distributions, a common procedure in signal processing. The result is a true 3-dimensional tomography, in which the localization of brain signals is preserved with a low amount of dispersion.
