# Supplementary material for: Combined treatment of nerve growth factor and transcranical direct current stimulations to improve outcome in children with vegetative state after out-of-hospital cardiac arrest
Source: Biol Direct. 2023 May 10;18:24. doi: 10.1186/s13062-023-00379-5 (PMC10170696; doi:10.1186/s13062-023-00379-5)
Supplement: Supplementary file 2 — Supplementary Material 2 [file 13062_2023_379_MOESM2_ESM.docx]

**Figures Legend of Supplementary Materials**

**eFigure 1. Modified Ashworth Scale**

The Modified Ashworth scale is a 6 point numerical scale that graded spasticity from 0 to 4: 0: No increase in muscle tone; 1: Slight increase in muscle tone, with a catch and release or minimal resistance at the end of the range of motion when an affected part(s) is moved in flexion or extension; 1+: Slight increase in muscle tone, manifested as a catch, followed by minimal resistance through the remainder (less than half) of the range of motion; 2: A marked increase in muscle tone throughout most of the range of motion, but affected part(s) are still easily moved; 3: Considerable increase in muscle tone, passive movement difficult; 4: Affected part(s) rigid in flexion or extension.

**eFigure 2. Gross Motor Function Measure**

The Gross Motor Function Measure (GMFM) consists of 88 items grouped into 5 dimensions: A (lying and rolling), B (sitting), C (crawling and kneeling), D (standing), E (walking, running and jumping). Scores for each dimension are expressed as a percentage of the maximum score for that dimension. A total score is obtained by adding the scores for all dimensions and dividing by 5, ranging from 0 to 100.

**eFigure 3. Gross Motor Function Measure (GMFM) score sheet.**

The Gross Motor Function Classification System (GMFCS) is used to classify each child's level of gross motor function with skill levels from I to V.

**eFigure 4. Disability Rating Scale**

The Disability Rating Scale (DRS) provides insight into the patient’s cognitive impairment by tracking progress over time. It has 8 items that address the three original World Health Organization categories of “impairment”, “disability” and “handicap”. The maximum score a patient can obtain on the DRS is 29 (extreme vegetative state). A person without disability would score zero. The DRS if fairly easy to use and can generally be completed in under 5 minutes.
