# Supplementary material for: Combined treatment of nerve growth factor and transcranical direct current stimulations to improve outcome in children with vegetative state after out-of-hospital cardiac arrest
Source: Biol Direct. 2023 May 10;18:24. doi: 10.1186/s13062-023-00379-5 (PMC10170696; doi:10.1186/s13062-023-00379-5)
Supplement: Supplementary file 3 — Supplementary Material 3 [file 13062_2023_379_MOESM3_ESM.pdf]

GROSS MOTOR FUNCTION MEASURE (GMFM)
SCORE SHEET (GMFM-88 and GMFM-66 scoring)

Child's Name: ______________________ ID#: ______________________

Assessment Date: ______________________
year / month / day

GMFCS Level[1]:

☐ I ☐ II ☐ III ☐ IV ☐ V

Date of Birth: ______________________
year / month / day

Chronological Age: ______________________
year / month / day

Evaluator's Name: ______________________

Testing Condition (e.g., room, clothing, time, others present):

____

The GMFM is a standardized observational instrument designed and validated to measure change in gross motor function over time in children with cerebral palsy. The scoring key is meant to be a general guideline. However, most of the items have specific descriptors for each score. It is imperative that the guidelines contained in the manual be used for scoring each item.

SCORING KEY

0 = does not initiate
1 = initiates
2 = partially completes
3 = completes
9 (or leave blank) = not tested (NT) [used for the GMAE-2 scoring*]

It is important to differentiate a true score of "0" (child does not initiate) from an item which is Not Tested (NT) if you are interested in using the GMFM-66 Ability Estimator (GMAE) Software.

*The GMAE-2 software is available for downloading from www.canchild.ca for those who have purchased the GMFM manual. The GMFM-66 is only valid for use with children who have cerebral palsy.

Contact for Research Group:
CanChild Centre for Childhood Disability Research,
Institute for Applied Health Sciences, McMaster University,
1400 Main St. W., Room 408,
Hamilton, ON Canada L8S 1C7
Email: canchild@mcmaster.ca Website: www.canchild.ca

[1]GMFCS level is a rating of severity of motor function. Definitions for the GMFCS-E&R (expanded & revised) are found in Palisano et al. (2008). Developmental Medicine & Child Neurology. 50:744-750 and in the GMAE-2 scoring software. http://motorgrowth.canchild.ca/en/GMFCS/resources/GMFCS-ER.pdf

© 2013 Dianne Russell and Peter Rosenbaum, McMaster University. All rights reserved.

Check (3) the appropriate score: if an item is not tested (NT), circle the item number on the right column

	Item	A: LYING & ROLLING	SCORE				NT
	1.	SUP, HEAD IN MIDLINE: TURNS HEAD WITH EXTREMITIES SYMMETRICAL	0☐	1☐	2☐	3☐	1.
*	2.	SUP: BRINGS HANDS TO MIDLINE, FINGERS ONE WITH THE OTHER	0☐	1☐	2☐	3☐	2.
	3.	SUP: LIFTS HEAD 45°	0☐	1☐	2☐	3☐	3.
	4.	SUP: FLEXES R HIP & KNEE THROUGH FULL RANGE	0☐	1☐	2☐	3☐	4.
	5.	SUP: FLEXES L HIP & KNEE THROUGH FULL RANGE	0☐	1☐	2☐	3☐	5.
*	6.	SUP: REACHES OUT WITH R ARM, HAND CROSSES MIDLINE TOWARD TOY	0☐	1☐	2☐	3☐	6.
*	7.	SUP: REACHES OUT WITH L ARM, HAND CROSSES MIDLINE TOWARD TOY	0☐	1☐	2☐	3☐	7.
	8.	SUP: ROLLS TO PR OVER R SIDE	0☐	1☐	2☐	3☐	8.
	9.	SUP: ROLLS TO PR OVER L SIDE	0☐	1☐	2☐	3☐	9.
*	10.	PR: LIFTS HEAD UPRIGHT	0☐	1☐	2☐	3☐	10.
	11.	PR ON FOREARMS: LIFTS HEAD UPRIGHT, ELBOWS EXT., CHEST RAISED	0☐	1☐	2☐	3☐	11.
	12.	PR ON FOREARMS: WEIGHT ON R FOREARM, FULLY EXTENDS OPPOSITE ARM FORWARD	0☐	1☐	2☐	3☐	12.
	13.	PR ON FOREARMS: WEIGHT ON L FOREARM, FULLY EXTENDS OPPOSITE ARM FORWARD	0☐	1☐	2☐	3☐	13.
	14.	PR: ROLLS TO SUP OVER R SIDE	0☐	1☐	2☐	3☐	14.
	15.	PR: ROLLS TO SUP OVER L SIDE	0☐	1☐	2☐	3☐	15.
	16.	PR: PIVOTS TO R 90° USING EXTREMITIES	0☐	1☐	2☐	3☐	16.
	17.	PR: PIVOTS TO L 90° USING EXTREMITIES	0☐	1☐	2☐	3☐	17.
		**TOTAL DIMENSION A**					

	Item	B: SITTING	SCORE				NT
*	18.	SUP, HANDS GRASPED BY EXAMINER: PULLS SELF TO SITTING WITH HEAD CONTROL	0☐	1☐	2☐	3☐	18.
	19.	SUP: ROLLS TO R SIDE, ATTAINS SITTING	0☐	1☐	2☐	3☐	19.
	20.	SUP: ROLLS TO L SIDE, ATTAINS SITTING	0☐	1☐	2☐	3☐	20.
*	21.	SIT ON MAT, SUPPORTED AT THORAX BY THERAPIST: LIFTS HEAD UPRIGHT, MAINTAINS 3 SECONDS	0☐	1☐	2☐	3☐	21.
*	22.	SIT ON MAT, SUPPORTED AT THORAX BY THERAPIST: LIFTS HEAD MIDLINE, MAINTAINS 10 SECONDS	0☐	1☐	2☐	3☐	22.
*	23.	SIT ON MAT, ARM(S) PROPPING: MAINTAINS, 5 SECONDS	0☐	1☐	2☐	3☐	23.
*	24.	SIT ON MAT: MAINTAIN, ARMS FREE, 3 SECONDS	0☐	1☐	2☐	3☐	24.
*	25.	SIT ON MAT WITH SMALL TOY IN FRONT: LEANS FORWARD, TOUCHESTOY, RE-ERECTS WITHOUT ARM PROPPING	0☐	1☐	2☐	3☐	25.
*	26.	SIT ON MAT: TOUCHES TOY PLACED 45° BEHIND CHILD'S R SIDE, RETURNS TO START	0☐	1☐	2☐	3☐	26.
*	27.	SIT ON MAT: TOUCHES TOY PLACED 45° BEHIND CHILD'S L SIDE, RETURNS TO START	0☐	1☐	2☐	3☐	27.
	28.	R SIDE SIT: MAINTAINS, ARMS FREE, 5 SECONDS	0☐	1☐	2☐	3☐	28.
	29.	L SIDE SIT: MAINTAINS, ARMS FREE, 5 SECONDS	0☐	1☐	2☐	3☐	29.
*	30.	SIT ON MAT: LOWERS TO PR WITH CONTROL	0☐	1☐	2☐	3☐	30.
*	31.	SIT ON MAT WITH FEET IN FRONT: ATTAINS 4 POINT OVER R SIDE	0☐	1☐	2☐	3☐	31.
*	32.	SIT ON MAT WITH FEET IN FRONT: ATTAINS 4 POINT OVER L SIDE	0☐	1☐	2☐	3☐	32.
	33.	SIT ON MAT: PIVOTS 90°, WITHOUT ARMS ASSISTING	0☐	1☐	2☐	3☐	33.
*	34.	SIT ON BENCH: MAINTAINS, ARMS AND FEET FREE, 10 SECONDS	0☐	1☐	2☐	3☐	34.
*	35.	STD: ATTAINS SIT ON SMALL BENCH	0☐	1☐	2☐	3☐	35.
*	36.	ON THE FLOOR: ATTAINS SIT ON SMALL BENCH	0☐	1☐	2☐	3☐	36.
*	37.	ON THE FLOOR: ATTAINS SIT ON LARGE BENCH	0☐	1☐	2☐	3☐	37.
		**TOTAL DIMENSION B**					

© 2013 Dianne Russell and Peter Rosenbaum, McMaster University. All rights reserved.

Item		C: CRAWLING & KNEELING	SCORE				NT
	38.	PR: CREEPS FORWARD 1.8m (6')	0☐	1☐	2☐	3☐	38.
*	39.	4 POINT: MAINTAINS, WEIGHT ON HANDS AND KNEES, 10 SECONDS	0☐	1☐	2☐	3☐	39.
*	40.	4 POINT: ATTAINS SIT ARMS FREE	0☐	1☐	2☐	3☐	40.
*	41.	PR: ATTAINS 4 POINT, WEIGHT ON HANDS AND KNEES	0☐	1☐	2☐	3☐	41.
*	42.	4 POINT: REACHES FORWARD WITH R ARM, HAND ABOVE SHOULDER LEVEL	0☐	1☐	2☐	3☐	42.
*	43.	4 POINT: REACHES FORWARD WITH L ARM, HAND ABOVE SHOULDER LEVEL	0☐	1☐	2☐	3☐	43.
*	44.	4 POINT: CRAWLS OR HITCHES FORWARD 1.8m(6')	0☐	1☐	2☐	3☐	44.
*	45.	4 POINT: CRAWLS RECIPROCALLY FORWARD1.8m (6')	0☐	1☐	2☐	3☐	45.
*	46.	4 POINT: CRAWLS UP 4 STEPS ON HANDS AND KNEES/FEET	0☐	1☐	2☐	3☐	46.
	47.	4 POINT: CRAWLS BACKWARDS DOWN 4 STEPS ON HANDS AND KNEES/FEET	0☐	1☐	2☐	3☐	47.
*	48.	SIT ON MAT: ATTAINS HIGH KN USING ARMS, MAINTAINS, ARMS FREE, 10 SECONDS	0☐	1☐	2☐	3☐	48.
	49.	HIGH KN: ATTAINS HALF KN ON R KNEE USING ARMS, MAINTAINS, ARMS FREE, 10 SECONDS	0☐	1☐	2☐	3☐	49.
	50.	HIGH KN: ATTAINS HALF KN ON L KNEE USING ARMS, MAINTAINS, ARMS FREE, 10 SECONDS	0☐	1☐	2☐	3☐	50.
*	51.	HIGH KN: KN WALKS FORWARD 10 STEPS, ARMS FREE	0☐	1☐	2☐	3☐	51.

TOTAL DIMENSION C ☐

Item		D: STANDING	SCORE				NT
*	52.	ON THE FLOOR: PULLS TO STD AT LARGE BENCH	0☐	1☐	2☐	3☐	52.
*	53.	STD: MAINTAINS, ARMS FREE, 3 SECONDS	0☐	1☐	2☐	3☐	53.
*	54.	STD: HOLDING ON TO LARGE BENCH WITH ONE HAND, LIFTS R FOOT, 3 SECONDS	0☐	1☐	2☐	3☐	54.
*	55.	STD: HOLDING ON TO LARGE BENCH WITH ONE HAND, LIFTS L FOOT, 3 SECONDS	0☐	1☐	2☐	3☐	55.
*	56.	STD: MAINTAINS, ARMS FREE, 20 SECONDS	0☐	1☐	2☐	3☐	56.
*	57.	STD: LIFTS L FOOT, ARMS FREE, 10 SECONDS	0☐	1☐	2☐	3☐	57.
*	58.	STD: LIFTS R FOOT, ARMS FREE, 10 SECONDS	0☐	1☐	2☐	3☐	58.
*	59.	SIT ON SMALL BENCH: ATTAINS STD WITHOUT USING ARMS	0☐	1☐	2☐	3☐	59.
*	60.	HIGH KN: ATTAINS STD THROUGH HALF KN ON R KNEE, WITHOUT USING ARMS	0☐	1☐	2☐	3☐	60.
*	61.	HIGH KN: ATTAINS STD THROUGH HALF KN ON L KNEE, WITHOUT USING ARMS	0☐	1☐	2☐	3☐	61.
*	62.	STD: LOWERS TO SIT ON FLOOR WITH CONTROL, ARMS FREE	0☐	1☐	2☐	3☐	62.
*	63.	STD: ATTAINS SQUAT, ARMS FREE	0☐	1☐	2☐	3☐	63.
*	64.	STD: PICKS UP OBJECT FROM FLOOR, ARMS FREE, RETURNS TO STAND	0☐	1☐	2☐	3☐	64.

TOTAL DIMENSION D ☐

© 2013 Dianne Russell and Peter Rosenbaum, McMaster University. All rights reserved.

Item		E: WALKING, RUNNING & JUMPING	SCORE				NT
*	65.	STD, 2 HANDS ON LARGE BENCH: CRUISES 5 STEPS TO R	0☐	1☐	2☐	3☐	65.
*	66.	STD, 2 HANDS ON LARGE BENCH: CRUISES 5 STEPS TO L	0☐	1☐	2☐	3☐	66.
*	67.	STD, 2 HANDS HELD: WALKS FORWARD 10 STEPS	0☐	1☐	2☐	3☐	67.
*	68.	STD, 1 HAND HELD: WALKS FORWARD 10 STEPS	0☐	1☐	2☐	3☐	68.
*	69.	STD: WALKS FORWARD 10 STEPS	0☐	1☐	2☐	3☐	69.
*	70.	STD: WALKS FORWARD 10 STEPS, STOPS, TURNS 180°, RETURNS	0☐	1☐	2☐	3☐	70.
*	71.	STD: WALKS BACKWARD 10 STEPS	0☐	1☐	2☐	3☐	71.
*	72.	STD: WALKS FORWARD 10 STEPS, CARRYING A LARGE OBJECT WITH 2 HANDS	0☐	1☐	2☐	3☐	72.
*	73.	STD: WALKS FORWARD 10 CONSECUTIVE STEPS BETWEEN PARALLEL LINES 20cm (8")APART	0☐	1☐	2☐	3☐	73.
*	74.	STD: WALKS FORWARD 10 CONSECUTIVE STEPS ON A STRAIGHT LINE 2cm (3/4") WIDE	0☐	1☐	2☐	3☐	74.
*	75.	STD: STEPS OVER STICK AT KNEE LEVEL, R FOOT LEADING	0☐	1☐	2☐	3☐	75.
*	76.	STD: STEPS OVER STICK AT KNEE LEVEL, L FOOT LEADING	0☐	1☐	2☐	3☐	76.
*	77.	STD: RUNS 4.5m (15'), STOPS & RETURNS	0☐	1☐	2☐	3☐	77.
*	78.	STD: KICKS BALL WITH R FOOT	0☐	1☐	2☐	3☐	78.
*	79.	STD: KICKS BALL WITH L FOOT	0☐	1☐	2☐	3☐	79.
*	80.	STD: JUMPS 30cm (12") HIGH, BOTH FEET SIMULTANEOUSLY	0☐	1☐	2☐	3☐	80.
*	81.	STD: JUMPS FORWARD 30 cm (12"), BOTH FEET SIMULTANEOUSLY	0☐	1☐	2☐	3☐	81.
*	82.	STD ON R FOOT: HOPS ON R FOOT 10 TIMES WITHIN A 60cm (24") CIRCLE	0☐	1☐	2☐	3☐	82.
*	83.	STD ON L FOOT: HOPS ON L FOOT 10 TIMES WITHIN A 60cm (24") CIRCLE	0☐	1☐	2☐	3☐	83.
*	84.	STD, HOLDING 1 RAIL: WALKS UP 4 STEPS, HOLDING 1 RAIL, ALTERNATING FEET	0☐	1☐	2☐	3☐	84.
*	85.	STD, HOLDING 1 RAIL: WALKS DOWN 4 STEPS, HOLDING 1 RAIL, ALTERNATING FEET	0☐	1☐	2☐	3☐	85.
*	86.	STD: WALKS UP 4 STEPS, ALTERNATING FEET	0☐	1☐	2☐	3☐	86.
*	87.	STD: WALKS DOWN 4 STEPS, ALTERNATING FEET	0☐	1☐	2☐	3☐	87.
*	88.	STD ON 15cm (6") STEP: JUMPS OFF, BOTH FEET SIMULTANEOUSLY	0☐	1☐	2☐	3☐	88.

TOTAL DIMENSION E ☐

Was this assessment indicative of this child's "regular" performance? YES ☐ NO ☐

COMMENTS:

© 2013 Dianne Russell and Peter Rosenbaum, McMaster University. All rights reserved.

GMFM-88 SUMMARY SCORE

DIMENSION	CALCULATION OF DIMENSION % SCORES				GOAL AREA (indicated with ✓ check)
A. Lying & Rolling	Total Dimension A / 51	= ____ / 51	× 100 =	____ %	A. ☐
B. Sitting	Total Dimension B / 60	= ____ / 60	× 100 =	____ %	B. ☐
C. Crawling & Kneeling	Total Dimension C / 42	= ____ / 42	× 100 =	____ %	C. ☐
D. Standing	Total Dimension D / 39	= ____ / 39	× 100 =	____ %	D. ☐
E. Walking, Running & Jumping	Total Dimension E / 72	= ____ / 72	× 100 =	____ %	E. ☐

TOTAL SCORE = $\frac{\%A + \%B + \%C + \%D + \%E}{\text{Total \# of Dimensions}}$

= $\frac{\_\_\_\_\_\_\_\_}{5}$ = ________ = ________ %

GOAL TOTAL SCORE = $\frac{\text{Sum of \%scores for each dimension identified as a goal area}}{\text{\# of Goal areas}}$

= ________ = ________ %

GMFM-66 Gross Motor Ability Estimator Score [1]

GMFM-66 Score = ________ ________ to ________
95% Confidence Intervals

previous GMFM-66 Score = ________ ________ to ________
95% Confidence Intervals

change in GMFM-66 = ________

[1] from the Gross Motor Ability Estimator (GMAE-2) Software

© 2013 Dianne Russell and Peter Rosenbaum, McMaster University. All rights reserved.

TESTING WITH AIDS/ORTHOSES USING THE GMFM-88

Indicate below with a check (4) which aid/orthosis was used and what dimension it was first applied. (There may be more than one).

AID		Dimension
Rollator/pusher	☐	________
Walker	☐	________
H Frame crutches	☐	________
Crutches	☐	________
Quad Cane	☐	________
Cane	☐	________
None	☐	________
Other ______________________ (please specify)	☐	________

Orthosis		Dimension
Hip Control	☐	________
Knee Control	☐	________
Ankle-foot Control	☐	________
Foot Control	☐	________
Shoes	☐	________
None	☐	________
Other ______________________ (please specify)	☐	________

GMFM-88 SUMMARY SCORE USING AIDS/ORTHOSES

DIMENSION	CALCULATION OF DIMENSION % SCORES	GOAL AREA (indicated with ✓ check)
F. Lying & Rolling	$\frac{\text{Total Dimension A}}{51} = \frac{\_\_\_\_}{51} \times 100 = \_\_\_\_ \%$	A. ☐
G. Sitting	$\frac{\text{Total Dimension B}}{60} = \frac{\_\_\_\_}{60} \times 100 = \_\_\_\_ \%$	B. ☐
H. Crawling & Kneeling	$\frac{\text{Total Dimension C}}{42} = \frac{\_\_\_\_}{42} \times 100 = \_\_\_\_ \%$	C. ☐
I. Standing	$\frac{\text{Total Dimension D}}{39} = \frac{\_\_\_\_}{39} \times 100 = \_\_\_\_ \%$	D. ☐
J. Walking, Running & Jumping	$\frac{\text{Total Dimension E}}{72} = \frac{\_\_\_\_}{72} \times 100 = \_\_\_\_ \%$	E. ☐

TOTAL SCORE = $\frac{\%A + \%B + \%C + \%D + \%E}{\text{Total \# of Dimensions}}$

= $\frac{\_\_\_\_\_\_\_\_}{5}$ = ________ = ________ %

GOAL TOTAL SCORE = $\frac{\text{Sum of \%scores for each dimension identified as a goal area}}{\text{\# of Goal areas}}$

= ________________ = ________ %

© 2013 Dianne Russell and Peter Rosenbaum, McMaster University. All rights reserved.
