# Supplementary figures and images for: Combined treatment of nerve growth factor and transcranical direct current stimulations to improve outcome in children with vegetative state after out-of-hospital cardiac arrest
Source: Biol Direct. 2023 May 10;18:24. doi: 10.1186/s13062-023-00379-5 (PMC10170696; doi:10.1186/s13062-023-00379-5)

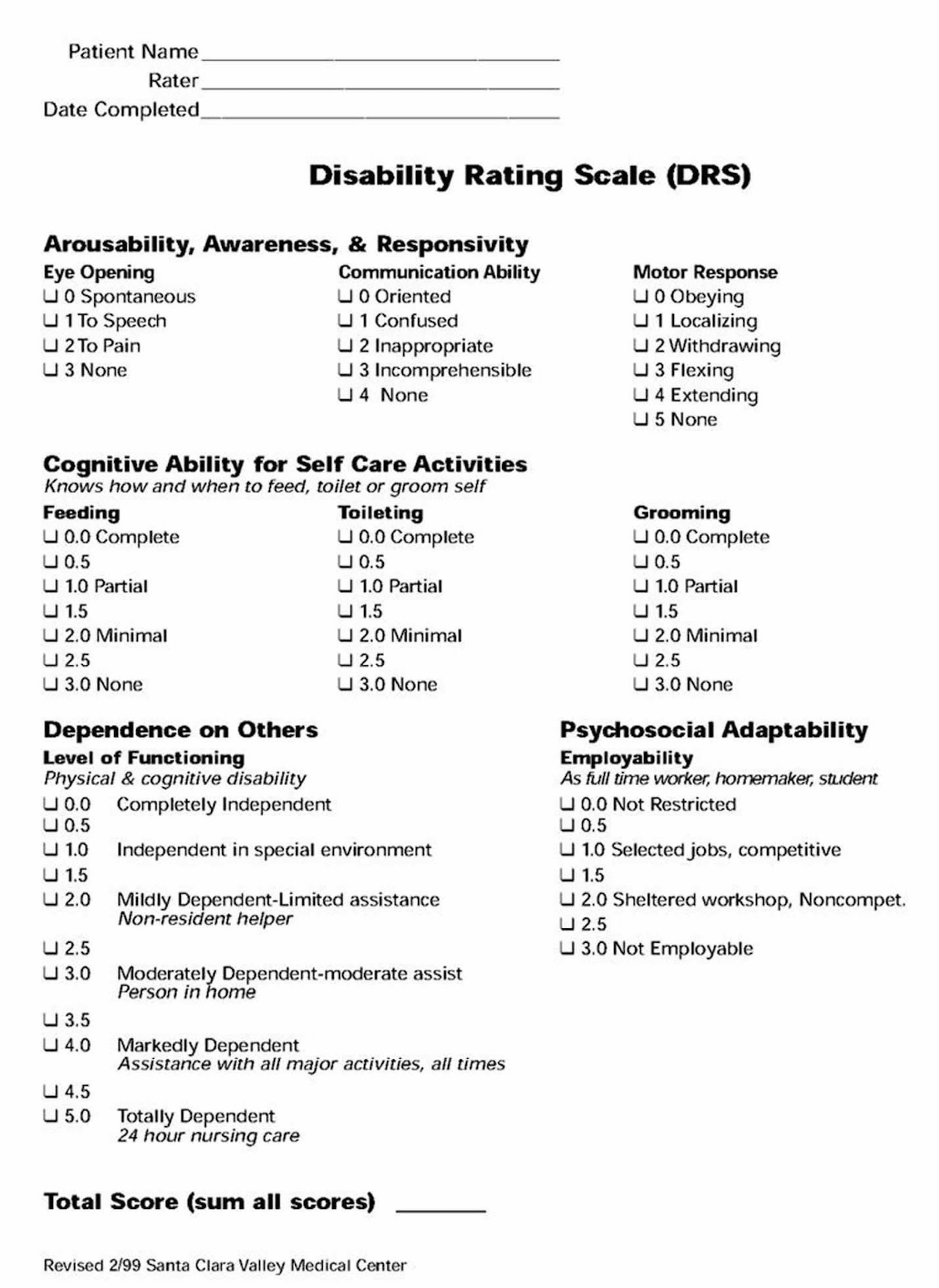

Supplement: Supplementary file 4 — Supplementary Material 4 [file 13062_2023_379_MOESM4_ESM.jpg]

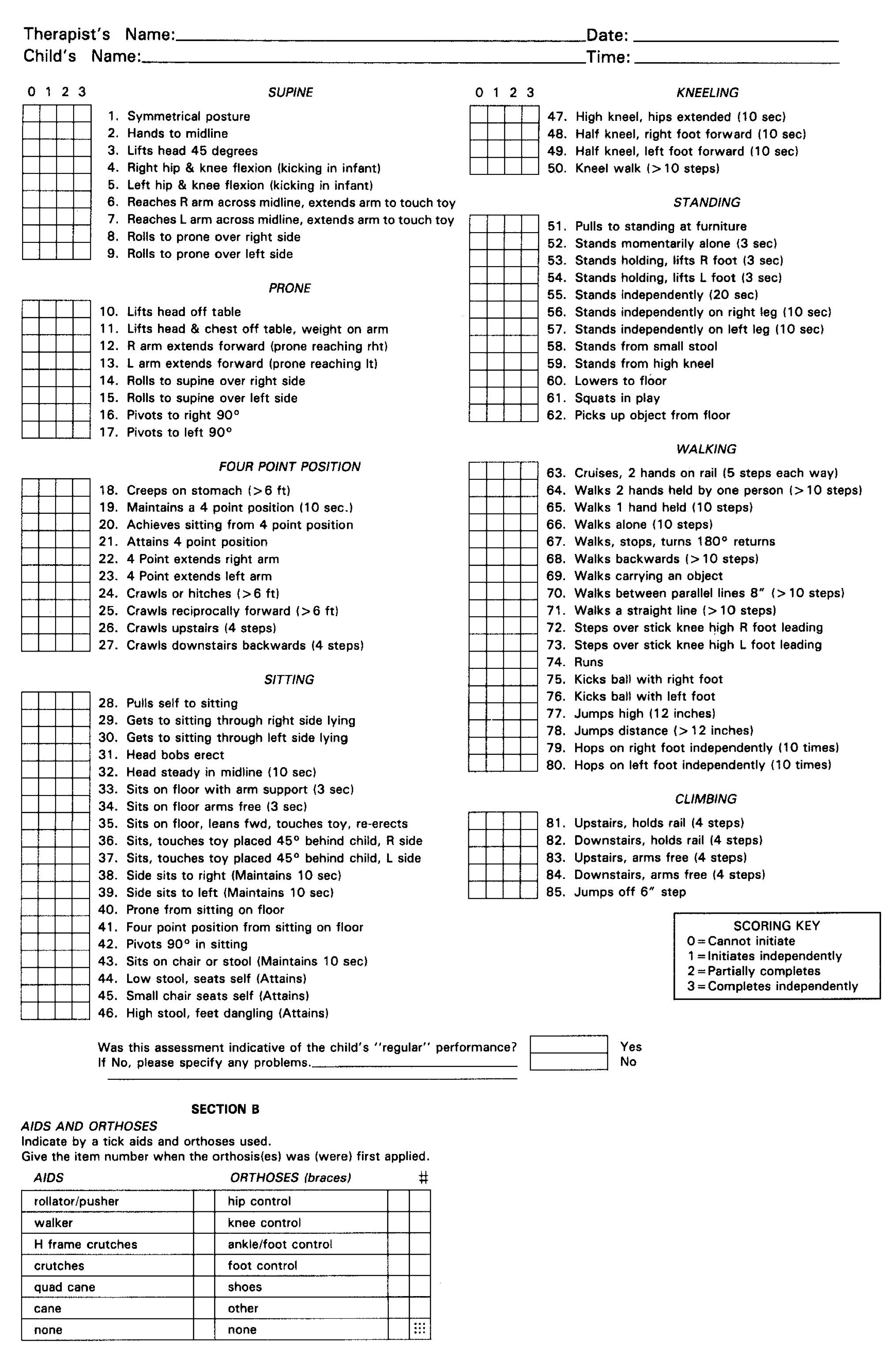

Supplement: Supplementary file 5 — Supplementary Material 5 [file 13062_2023_379_MOESM5_ESM.jpg]

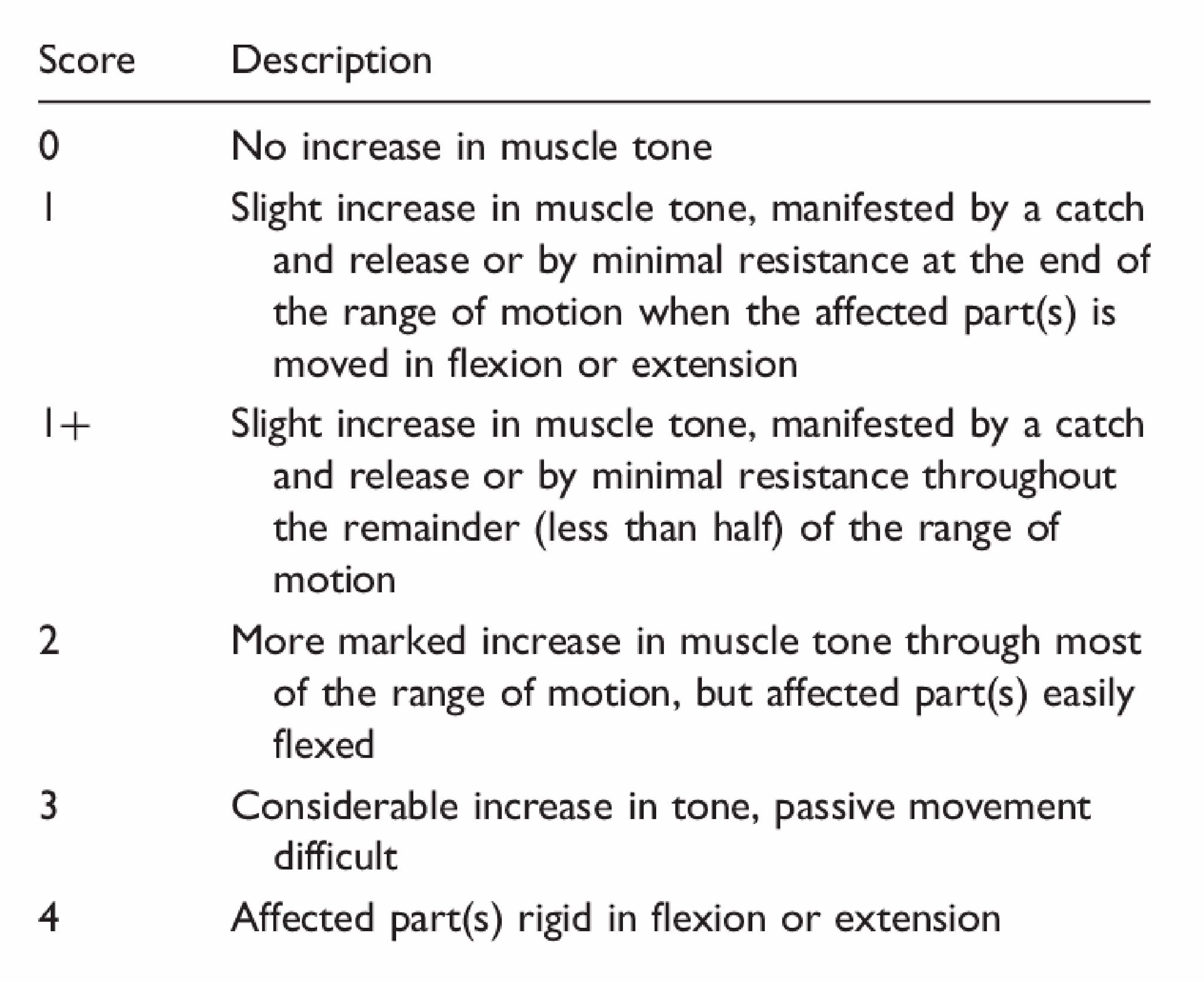

Supplement: Supplementary file 6 — Supplementary Material 6 [file 13062_2023_379_MOESM6_ESM.jpg]
